# 3D auxetic single material periodic structure with ultra-wide tunable bandgap

**DOI:** 10.1038/s41598-018-19963-1

**Published:** 2018-02-02

**Authors:** Luca D’Alessandro, Valentina Zega, Raffaele Ardito, Alberto Corigliano

**Affiliations:** Politecnico di Milano, Civil and Environmental Engineering, Milan, Italy

## Abstract

The design and the combination of innovative metamaterials are attracting increasing interest in the scientific community because of their unique properties that go beyond the ones of natural materials. In particular, auxetic materials and phononic crystals are widely studied for their negative Poisson’s ratio and their bandgap opening properties, respectively. In this work, auxeticity and phononic crystals bandgap properties are properly combined to obtain a single phase periodic structure with a tridimensional wide tunable bandgap. When an external tensile load is applied to the structure, the auxetic unit cells change their configurations by exploiting the negative Poisson’s ratio and this results in the tuning, either hardening or softening, of the frequencies of the modes limiting the 3D bandgap. Moreover, the expansion of the unit cell in all the directions, due to the auxeticity property, guarantees a fully 3D bandgap tunability of the proposed structure. Numerical simulations and analytical models are proposed to prove the claimed properties. The first experimental evidence of the tunability of a wide 3D bandgap is then shown thanks to the fabrication of a prototype by means of additive manufacturing.

## Introduction

In the recent years a strong attention has been devoted to metamaterials^1,2^ by the scientific community, due to the possibility of creating devices with unprecedented engineered properties, starting from electro-magnetic features to arrive more recently to the acoustic and elastic counterpart. Among the others, phononic crystals (PnCs)^3^ and auxetic metamaterials^4^ are gaining great interest, mainly because of their properties of controlling elastic wave propagation and of showing negative Poisson’s ratio, respectively.

Phononic crystals are periodic structures that may exhibit frequency ranges, called bandgaps, over which the transmission of acoustic and elastic waves is impeded. Bandgap width, frequency level, modal location and effective direction or isotropy of a PnC mainly depend on the geometry, topology and constitutive material properties^5^ of its unit cell. The basic features of the unit cell of a PnC can therefore be optimized in terms of shape^6^, material and mechanical characteristics to achieve particular bandgap properties such as bandgap at low frequency range^7–12^ or maximum relative bandgap width^13–16^. On the other hand, auxetic materials present very interesting features^17–21^ originating from negative Poisson’s ratio, such as increased shear modulus, indentation resistance, fracture toughness, energy absorption, porosity/permeability variation with strain and synclastic curvature, which also depend on the topology of the unit cell. It is therefore very interesting to study the combination of such properties^22–25^ to obtain a metamaterial endowed with controllable phononic bandgaps^26,27^ to be tailored, degraded or enhanced during its functioning. Several tunable PnCs,^28,29^ which take advantage of different materials or properties combined together, are available. Auxetic materials, for example, are used in PnCs in combination with conventional cores^30^, with local resonators^31^ or with distributed shunted piezoelectric patches^32^ to enhance the effective Young’s modulus and to lower the frequencies limiting the bandgap^33,34^. Although single-phase, 3D tunable PnC structures are of great interest for the full control of 3D wave propagation and manufacturing purposes, few are the examples in the literature: tunable 3D PnCs are numerically studied^35–39^ while experimental evidence is reported only for the 2D case^40,41^.

In this work, a 3D single-phase PnC structure endowed with ultra-wide complete 3D bandgaps is proposed. The tunability of the first bandgap is obtained by exploiting the negative Poisson’s ratio of its unit cells, whose topology is a mix of oustanding PnC properties^15^ and 3D-extension of the results of a proper topology optimization on the auxetic behaviour^42,43^. In the first part of the paper, numerical simulations are adopted to prove the bangap tuning as a strict consequence of the expansion in all the orthogonal directions of the auxetic unit cell. Moreover, simple analytical models are shown to gain insight into the mechanical behaviour that is behind the tuning. A prototype of 3 × 3 × 3 unit cells is fabricated in NylonPA 12^44^ by means of additive manufacturing and tested to asses the transmission spectra for different levels of load. A good agreement between experimental and numerical results, based on a Standard Linear Solid visco-elastic model^45,46^, is reached.

## Results

### Unit cell analysis

**Figure 1:**
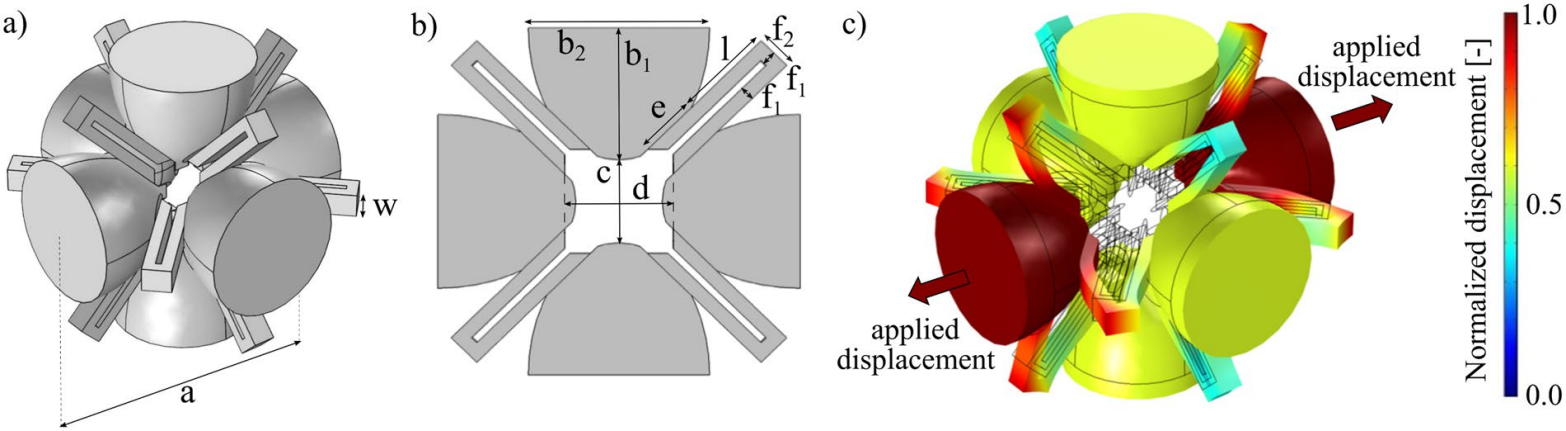
Unit cell topology. (**a**) 3D representation of the unit cell. (**b**) 2D cross section with respect to one of the principal planes. c) Auxetic (*v* ≈ − 0.6) deformed shape of the unit cell computed by means of the Solid Mechanics Module of COMSOL Multiphysics v5.3. The contour of the displacement magnitude field is shown in color.

The 3D simple cubic unit cell of the proposed structure is shown in Fig. 1a, while a 2D cross section with respect to one of its principal planes of symmetry is depicted in Fig. 1b. The unit cell structure is characterised by ellipsoides connected to each other by U-shaped elements. In the following, the ellipsoides will be referred to as ‘masses’, since they are the most rigid parts of the unit cell, while the U-shaped elements as ‘elastic connections’ due to their connecting function. The geometric dimensions shown in Fig. 1a,b are reported in Table 1, where *a* is the unit cell characteristic dimension.

**Table 1:** Geometric dimensions of unit cell components described in Fig. 1a,b.

Quantity	*w*	*b*_1_	*b*_2_	*c*	*d*	*e*	*f*_1_	*f*_2_	*l*
Dimension	0.08*a*	0.38*a*	0.50*a*	0.24*a*	0.30*a*	0.17*a*	0.04*a*	0.10*a*	0.27*a*

In Fig. 1c, the auxetic behaviour of the unit cell of the proposed structure is also reported: by applying two opposite unitary displacements to the semi-ellipsoides along one principal direction (see red arrows in Fig. 1c), the ellipsoides which lay on the plane orthogonal to the load move away from each other’s by more than half of the applied displacement, thus showing a negative Poisson’s ratio (*v* ≈ − 0.6). It is important to notice that, by proper design of the elastic connection, a Poisson’s ratio near to −1.0 can be reached^42,43^, thus resulting in a fully symmetric 3D behaviour. In this work the manufacturability constraints lead to the adopted design.

**Figure 2:**
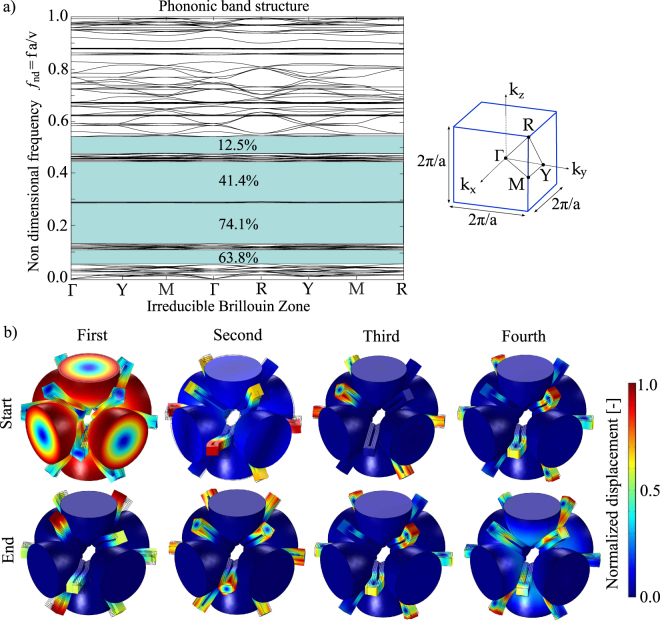
Dynamic analysis of the unit cell. (**a**) Phononic band structure with associated Irreducible Brillouin Zone (IBZ) of simple-cubic crystal structure (*f*_*nd*_ non dimensional frequency, *f* frequency, ${\rm{v}}=\sqrt{E/\rho }$ sound velocity in the material). (**b**) Modes shapes at each of the four bandgap extrema (total displacement normalized with respect to the maximum displacement).

The dynamic behaviour of the proposed periodic structure is described, as typical of PnCs, by means of the dispersion analysis performed over the unit cell through the use of Bloch’s periodic boundary conditions in COMSOL Multiphysics v5.3. In Fig. 2a, several wide 3D complete bandgaps are present while the modes at the extrema of each bandgap are depicted in Fig. 2b for the sake of clarity. The first bandgap is characterised by 63.8% gap to mid-gap ratio, with limiting non dimensional frequencies equal to 0.0567 and 0.1100. The non dimensional frequency *f*_*nd*_ is defined as the ratio between the product of the frequency *f* and the unit cell dimension *a* and the sound velocity in the material ${\rm{v}}=\sqrt{E/\rho }$ with *E* and *ρ* the Young’s modulus and the density of the material, respectively. The low level of the frequency that opens the bandgap is in agreement with the results of a previous study^15^, that is referred to a structure with a similar dyamic behavior, basically governed by modes separation. In the present case, the mode that defines the bottom limit of this bandgap is located in the symmetry point *R* and is characterised by the rigid rotation of the masses with respect to the principal axis of each ellipsoide and by the consequent flexural-torsional deformation of the elastic connections. Due to the large modal mass and the relatively low modal stiffness, this mode is located at low frequency. On the other side, the mode that defines the top limit of the first bandgap is located in the symmetry point Γ and is characterised by the in-plane (the reference plane is the one reported in Fig. 1b) flexural deformation of the elastic connections and by a negligible participation of the mass. The second passband is therefore characterised by flexural deformations of the elastic connections: in-plane modes are in the lower part of the frequency band, while out-of-plane modes are in the upper region, being the stiffness associated to the out-of-plane flexural deformation of such U-shaped beams higher than the corresponding in-plane one and the modal masses comparable.

**Figure 3:**
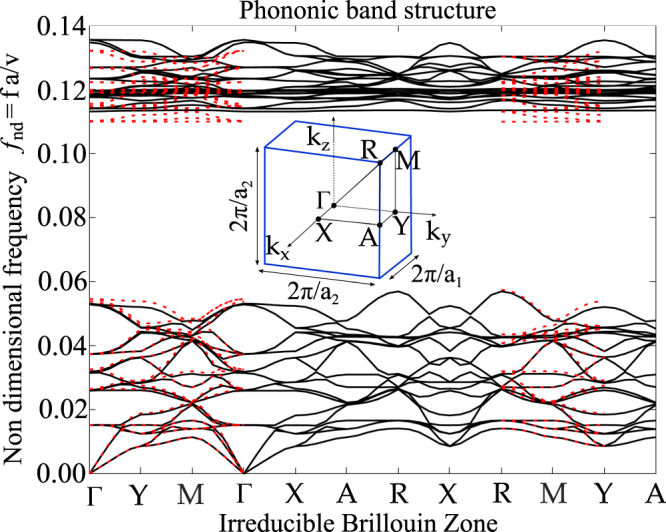
Dynamic analysis of the deformed unit cell. In red dotted line, the dispersion plot of the undeformed configuration shown in Fig. 2a. In solid black line, the dispersion plot of the deformed configuration of the unit cell (due to opposite applied displacements to ellipsoides in direction *x* equal to *q* = 0.005*a*) computed in COMSOL Multiphysics. The tetragonal IBZ for the deformed configuration is reported, where *a*_1_ = *a* + 2*q*, *a*_2_ = *a* + 1.2*q*. The same quantity *a* is used for frequency normalization.

When an external tensile force is applied on the two sides of the unit cell, it expands according to the deformed shape of Fig. 1c by exploiting its auxetic behaviour. Figure 3 reports in solid black line the phononic band structure of the first two passbands related to the deformed unit cell configuration, due to two opposite displacements equal to *q* = 0.005*a* applied on the ellipsoides in direction *x*. The considered Irreducible Brillouin Zone (IBZ) is the tetragonal one due to the non-symmetric (i.e. *ν* = − 0.6) auxetic deformation. In the figure, the band structure of the undeformed configuration (refer to Fig. 2a) is reported in red dashed line, superimposed to the deformed one only for the *k*-paths in common.The comparison of the different curves confirms the full 3D bandgap tuning, as better explained in the following.

**Figure 4:**
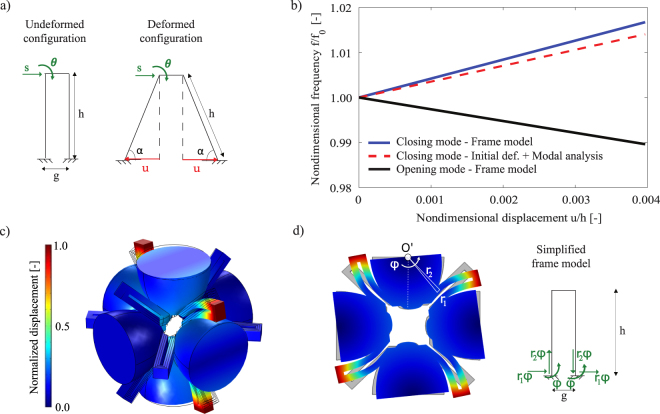
Simplified models for bandgap limiting modes. (**a**) Model for closing mode: schematic view of the undeformed and deformed configurations of the U-shaped elastic suspensions. (**b**) Frequency tuning of opening and closing modes in function of the *u* displacement induced by external tensile loads. The frequency in the undeformed and deformed configurations is indicated by *f*_0_ and *f* respectively. (**c**) Modal shape in point *Y* of the bottom limit of the first bandgap in the undeformed configuration. (**d**) Cross-sectional view of the modal shape of the bottom limit of the first bandgap and its simplified model.

The first bandgap tuning is characterised by a hardening behaviour of the upper limit, that involves all the symmetry points, thus resulting in a complete 3D bandgap widening. Conversely, the lower limit does not change in frequency in the entire IBZ path being the limiting mode located in the symmetry point *R*. Its modal stiffness is in fact characterised by the torsion of the elastic connections, which is not sensitive to the auxetic deformation. Nevertheless, the first bandgap experiences softening of the bottom limit mode band for the entire Γ − *Y* − *M* − Γ path. This is explained by analysing the modal shape in the point *Y* of this passband in the undeformed configuration (refer to Fig. 4c). This shape is characterised by rigid rotations of the ellipsoides along one of their axes: this movement is favoured by the deformed shape, thus resulting in a lower modal stiffness and consequently in a lower frequency.

To give a structural explanation of the frequency tuning of the second passband, the model of Fig. 4a,b is proposed. In Fig. 4a a schematic view of the elastic connections is reported both in the undeformed and deformed configurations: as the tensile load increases, the distance between the two arms of the U-shape increases, thus stiffening the in-plane flexural mode. This results in a shifting effect on the second passband and in an enhancing of the first bandgap width. A frame model is adopted to compute the stiffness of the in-plane flexural mode of the U-shaped elastic connections both in the undeformed and deformed configurations (see Fig. 4a). The frame is composed of two vertical beams of length *h* = *l* − *f*_1_/2 and one horizontal beam of length *g* = *f*_2_ − *f*_1_, both with in-plane thickness *f*_1_ and width *w*. Two degrees of freedom are considered: the horizontal displacement *s* and the rotation *θ* at the top left corner of the frame. The hypotheses are the infinite axial stiffness of the three beams, the use of the Euler Bernoulli beam model and the fixed boundary conditions at the bottom of the two vertical beams to mimic the ellipsoides not involved in the mode of interest. Because of these strong hypotheses, it does not pretend to be an exhaustive model, but it represents a good proof of the physical phenomenon responsible of the band gap tuning of the proposed structure. By considering an equal modal mass *m* for both the configurations studied and by applying the known relation between stiffness *k* and frequency *f* ($f=\sqrt{k/m}$), the variation of the frequency of the in-plane flexural mode of the elastic suspensions as function of the applied displacement *u* is obtained (see blue solid line in Fig. 4b). Note that *f*_0_ and *f* in Fig. 4b represent the frequencies of the mode of interest for the undeformed and deformed configurations respectively. The same curve is computed via the Solid Mechanics Module of COMSOL Multiphysics v5.3 through a modal analysis performed on the undeformed and deformed configurations respectively (see dashed line in Fig. 4b) and a good agreement is obtained. The small quantitative difference shown in Fig. 4b is mainly due to the hypotheses adopted.

The same model can be used to predict the softening behaviour of the opening mode. In that case, it is necessary to compute the elastic stiffness of the connections as a consequence of the rigid rotation of the masses, as shown in Fig. 4c. The frame model is subject to a set of imposed boundary conditions, see Fig. 4d, and the consequent reaction forces and moment are used to compute the overall moment around the axis of rotation. The same computation is repeated also in the deformed configuration, so that the frequency trend is obtained, see Fig. 4b. The softening behaviour is confirmed; moreover, the rate of reduction with respect to the imposed displacement is smaller than the rate of increase of the closing frequency, in agreement with the dispersion plot and with the experimental data presented in the next section.

### Finite structure description

**Figure 5:**
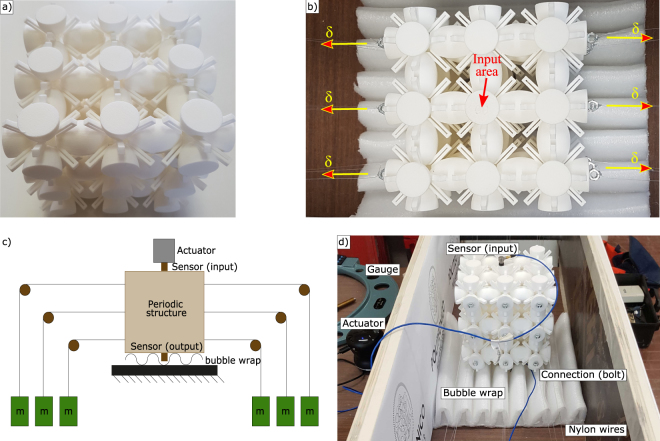
(**a**) Prototype fabricated by Selective Laser Sintering in NylonPA 12: 150 mm × 150 mm × 150 mm cube made of 3 × 3 × 3 repetitions of the unit cell of Fig. 1a. (**b**) Experimental setup: input area and directions of the applied displacement *δ* are highlighted, (**c**) schematic view of the experimental setup and of the loading mechanism employed to impose the displacement shown in Fig. 5b, (**d**) picture of the experimental setup (the actuator is removed to show the input sensor).

A prototype made of 3 × 3 × 3 unit cells of dimension *a* = 0.05 m is fabricated in NylonPA 12^44^ (*E* = 1586 MPa, *ν* = 0.4, *ρ* = 1000 kg/*m*^3^) by means of Selective Laser Sintering (see Fig. 5a). The dispersion plot, computed with a linear-elastic constitutive model and reported in dimensional terms in the Supplementary Figure s1, suggests that the first four bandgaps are contained in the range 0 − 20 kHz. The transmission spectrum is measured in the Γ − *Y* direction of the IBZ both in the undeformed and deformed configurations in order to catch the most interesting tuning phenomenon.

The number of cells adopted in the prototype is fully compatible with the features of the chosen method for rapid prototyping. On the other hand, there could be some doubt about the representation of the asymptotic behavior by means of such a finite structure. A specific clarification on this point is achieved by carrying out a set of parametric analyses, referred to an increasing number of layers in the propagation direction. The results of such analyses, reported in the Supplementary Figure s2, confirm that the chosen size of the prototype is sufficient to identify the correct bandgap width.

To acquire the spectrum in the undeformed configuration, the harmonic excitation is applied on the input area (see Fig. 5b) in the orthogonal direction with respect to the surface itself, while the output signal is acquired in the same direction, on the opposite face with respect to the input one. Tests are performed with a 60 s white noise from 0.2 kHz to 20.0 kHz. It is important to notice that with the applied experimental setup the maximum attenuation that can be measured is of 75 dB (i.e. 3.75 orders of magnitude).

**Figure 6:**
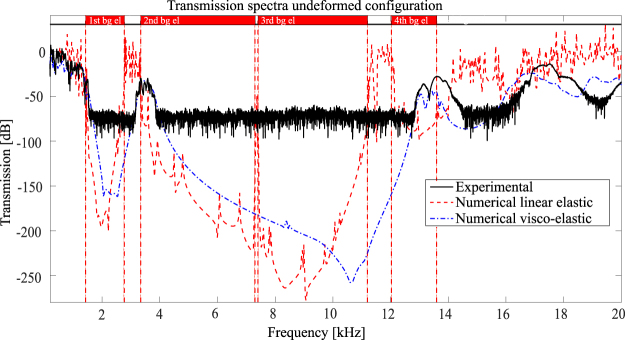
Transmission spectra in the undeformed configuration: experimental spectrum is in black solid line, numerical linear elastic in red dashed line and numerical visco-elastic with Standard Linear Solid model in blue dash-dot line. Vertical red dashed lines indicate the bandgap extrema for the linear elastic material behaviour.

The experimental transmission spectrum related to the undeformed configuration is reported in Fig. 6 in black solid line together with the numerical curve calculated by means of both the Solid Mechanics Module of COMSOL Multiphysics v5.3 and ABAQUS CAE v6.13 to double-check the results (see red dashed line in Fig. 6). A linear elastic model for the Nylon is adopted in the numerical simulation reported in Fig. 6: it is in good agreement with the bandgap extrema of Fig. 2a (vertical red dashed lines in Fig. 6), while some discrepancies with respect to the measured curve are experienced.

The first difference concerns the attenuation level in the bandgaps regions: while for the experimental curve it is fixed to the setup limit of 75 dB, the numerical curve predicts an attenuation up to 150 dB (7 orders of magnitude) in the first bandgap and 250 dB (12 orders of magnitude) in the third one. These values are considered reliable thanks to the double check with the finite element solvers. The second difference is the progressive shift in frequency and the damping of the experimental curve with respect to the numerical linear elastic one. A Standard Linear Solid visco-elastic model is then introduced in the numerical model with the only purpose of better catching the dynamic behaviour of the Nylon^45,46^ in the first two passbands, with main focus on the second one. The coefficients of the Maxwell’s branch are properly calibrated to describe the characteristics of the 3D-printed prototype in the first two passbands: a relaxation time equal to *τ*_*Maxwel*_ = 1.5306*e*−4 s and a Young’s modulus E _*Maxwel*_ = 490 MPa are then chosen^46^. The visco-elastic transmission spectrum is reported in Fig. 6 in blue dash-dot line. Thanks to the introduction of the visco-elastic model of the material, the numerical and the experimental curves are in good agreement in the first two passbands which represent the most interesting regions from the band gap tunability point of view. In order to properly catch the entire spectrum, a more refined visco-elastic model must be introduced, for instance by adding other Maxwell’s branches^46^.

An additional comment must be introduced regarding the third passband: in the phononic band structure of Fig. 1a it appears very narrow and by the modal analysis it results made by pure torsional deformations of the elastic connections with the elliptical masses that play a very limited role. Due to the torsional and local nature of these modes, they cannot be excited properly in standard transmission tests: this results in an attenuated peak in the numerical linear elastic analysis and even more damped one in the visco-elastic modelling. In the experimental curve it is not catched at all being the transmission beyond the measurable range (see Fig. 6 for more details).

**Figure 7:**
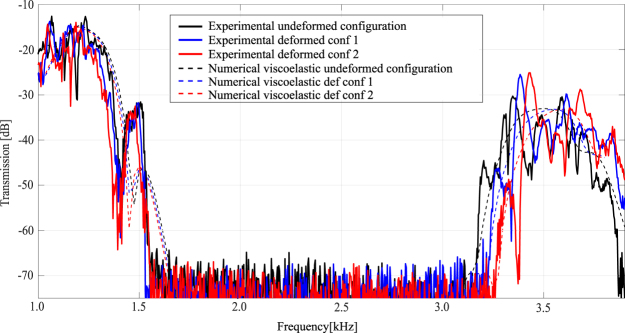
Tuning of the first bandgap: in solid lines the experimental results, in dashed lines the numerical visco-elastic model results. For configuration definition refer to Table 2.

The bandgap tunability of the finite prototype is experimentally tested by applying tensile horizontal forces on the two opposite faces, as indicated by the picture in Fig. 5b: to have a more precise control of the applied forces, each of the 18 semi-ellipsoides areas in the two faces is loaded autonomously, refer to the scheme presented in Fig. 5c. An overall view of the experimental setup is reported in Fig. 5d. Two steps of loading are considered (refer to Table 2): in the first step (configuration 1) the applied load per each ellipsoid-face is equal to 8 N and a displacement *δ* = 0.35 mm is obtained per each face; in the second step (configuration 2) the applied load per each ellipsoid-face is equal to 16 N and a displacement *δ* = 0.70 mm is obtained. The experimental transmission spectra of the undeformed and the two deformed configurations are reported in solid lines in Fig. 7. The bandgap limits are defined referring to the level of 40 dB of attenuation, further explaination can be found in the Supplementary Figure s3. Per each loading step, the bottom limit of the bandgap shifts backward while the top limit shifts forward, thus resulting in a bandgap widening as numerically predicted (refer to Fig. 4a).

**Table 2:** Load configurations and associated experimental bandgap tuning results.

Configuration	1	2
Load per each semi-ellipsoides face [N]	8.0 N	16.0 N
Resulting *δ* displacement (refer to Fig. 5b) [mm]	0.35 mm	0.70 mm
Ratio between imposed displacement $(\frac{2\delta }{3})$ and unit cell dimension *a* [%]	+0.47%	+0.93%
Experimental ${\rm{\Delta }}{f}_{start,{1}^{st}bg}$ (with respect to undeformed conf, at −40 dB) [%]	−0.9%	−2.1%
Experimental ${\rm{\Delta }}{f}_{end,{1}^{st}bg}$ (with respect to undeformed conf, at −40 dB) [%]	+ 1.7%	+ 3.2%
Experimental bandgap increase (${\rm{\Delta }}\frac{gap}{midgap}$ with respect to undeformed conf, at −40 dB) [%]	+ 3.1%	+ 6.2%

Quantitative data of the tuning experimental results are reported in Table 2: the expansion *δ* of the auxetic structure (refer to Fig. 5b) and the obtained experimental frequency shift of both bottom and top limits of the first bandgap are reported as function of the applied tensile load.

From Table 2 it appears that the frequency shift of the limits of the first bandgap occurs with a more favourable ratio than the one of the related expansion of the unit cell: in case of configuration 2, an applied displacement equal to the 0.93% of the unit cell dimension results in a bandgap width increase of the +6.2%. More specifically this hyper-tuning behaviour happens both for the mode at the lower limit of the bandgap, which experiences softening, and for the upper one, that presents hardening behaviour: the tuning widens, then, the bandgap in both directions. As a comparison, it is interesting to note that the models of Fig. 4 are able to predict the softening and hardening of the modes at the lower and upper limit of the bandgap, respectively, with good approximation. Moreover, the numerical dispersion analyses on the deformed unit cell shown in Fig. 4a well predict the real behaviour of the fabricated structure.

In Fig. 7 the spectra numerically computed per each configuration are also shown in dashed lines, the same colors refer to the same loading conditions. The numerical curves are obtained by means of the Solid Mechanics Module of COMSOL Multiphysics v5.3 by computing the spectrum after the solution of a non-linear static analysis with prescribed displacements along one direction. In order to mimic the experimental tests, a displacement along the wanted direction is applied on the 9-faces of the ellipsoides. More specifically the central of the 9-faces experiences also fixed displacements boundary conditions in the other two directions, while the remaining 8-faces are left free to expand. The numerical transmission curve is in excellent agreement with the experimental one in terms of tuning of the closing frequency of the bandgap (see Fig. 7), while it shows a slightly less prompt softening behaviour for the opening region. This inaccuracy of the numerical solution is mainly due to the choice of the visco-elastic model: the difference between the numerical and experimental curves is in fact the same also in the undeformed configuration where no tuning effects are present (see black curves in Fig. 7). In order to obtain a better fit, it is necessary to improve the visco-elastic model (i.e. to introduce more Maxwell’s branches), as already commented before for the transmission in the high frequency region.

## Discussion

The tunability of a wide complete 3D bandagap of a single-phase periodic structure is proved both numerically and experimentally. It represents the first experimental evidence of the tunability of a fully 3D wide bandgap of a single-phase periodic structure.

In the proposed periodic structure, the tunability of the 3D bandgap is achieved thanks to the expansion in the three orthogonal directions, due to the auxeticity of the unit cell: when an external tensile load/displacement is applied to two opposite faces of the finite structure, the unit cell configuration changes and the frequencies of the modes that define the bandgap experience either hardening or softening effects. The proposed working principle is also verified through a frame model of the elastic connections of the unit cells.

Experimental tests performed on a prototype of 3 × 3 × 3 unit cells fabricated in NylonPA 12 by means of additive manufacturing confirm the super-tunability properties of the proposed periodic structure: for an applied displacement of the order of 0.93% of the unit cell dimension, a tuning of the bandgap width, in terms of gap to mid-gap ratio, of the 6.2% is obtained. As previously stated, the experiment is carried out with propagation along the direction Γ − *Y*. To explore the 3D effect of bandgap tuning, as predicted by the dispersion plot in Fig. 3, the results of several simulations are considered, making use of the validated computational model. The transmission plots for different propagation paths are shown in the Supplementary Figures s4a–d.

The combination of auxetic and bandgap properties allows the design of a 3D tunable single phase periodic structures that can manage the elastic wave propagation in a full three-dimensional context.

## Methods

The prototype is fabricated by means of the Selective Laser Sintering technique, which permits the realization of any 3D geometry. More specifically this technique permits the realization of suspended structure through the sintering process thanks to the bearing capacity of the Nylon powder with respect to the sintered one.

To acquire the transmission spectra, the prototype is placed on a bubble wrap that isolates it from the environmental vibrations. A VibeTribe-Mamba with 20 W power and a frequency range from 40 Hz to 22 kHz is used as actuator, while two PCB Piezotronics 353 B 15 accelerometers, with sensitivity of 10 mV/g and resonant frequency of 70 kHz, are glued in the input and output surfaces respectively and are used as sensors. The data acquisition chain is completed with an 8-channel PCB 483 C 05 ICP Sensor Signal Conditioner, and a NI 9205 module, with 16-bit resolution. The acquired signals are sampled 51200 times per second and are post-processed by means of the Bartlett’s method. The signal is divided into 200 segments in order to guarantee a sufficient frequency resolution (i.e. *δf* = 3.3 Hz). The window function is the rectangular one.

To apply the tuning load, one Nylon wire is attached per each half-ellipsoid face in the tuning direction. The connection between the ellipsoid surface and the wire is defined via a light bolt, glued on the surface and tied and glued to the wire. The wire turns a *π*/4 angle around a pulley in order to have a vertical alignment. The tuning load is applied by attaching, to each wire, one calibrated weight, checked through a laboratory scale (tolerance of 1 g). The tuning effect is quantified through the measurement of the deformation induced on the structure: a gauge with 0.01 mm precision is employed for that purpose.

## Electronic supplementary material

Supplementary Information
