# Supplementary material for: 3D auxetic single material periodic structure with ultra-wide tunable bandgap
Source: Sci Rep. 2018 Feb 2;8:2262. doi: 10.1038/s41598-018-19963-1 (PMC5797155; doi:10.1038/s41598-018-19963-1)
Supplement: Supplementary file 1 — Supplementary Information [file 41598_2018_19963_MOESM1_ESM.pdf]

Supplementary Information for the manuscript:

3D auxetic single material periodic structure with ultra-wide tunable bandgap

Authors:

Luca D'Alessandro[1], Valentina Zega[1], Raffaele Ardito[1], Alberto Corigliano[1*]

[1]Politecnico di Milano, Civil and Environmental Engineering, Milan, Italy

*alberto.corigliano@polimi.it

1. Dispersion plot for the undeformed configuration

Dynamic analysis of the unit cell. Phononic band structure with associated Irreducible Brillouin Zone (IBZ) of simple-cubic crystal structure.

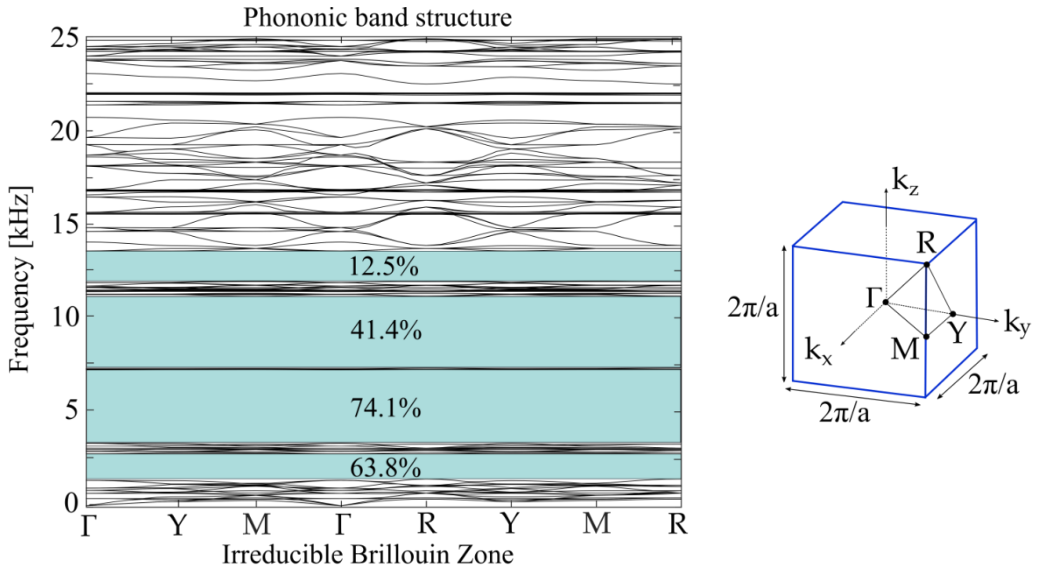

Figure s1: Dynamic analysis of the unit cell. Phononic band structure with associated Irreducible Brillouin Zone (IBZ) of simple-cubic crystal structure.

2. Transmission plot for periodic structures with an increasing number of layers in the propagation direction

The transmission plot for periodic structures with an increasing number of layers in the propagation direction (Fig. s2a)-c)) is shown in Fig. s2d). As the number of cells in the direction of propagation increases the attenuation increases. The choice of 3x3x3 cube is due to symmetric considerations and to fulfill the manufacturability constraints, together with to be able to measure the bandgap shift with sufficient reliability, knowing that the experimental setup limit is 75 dB of attenuation between input and output (thick black line in Figure s2).

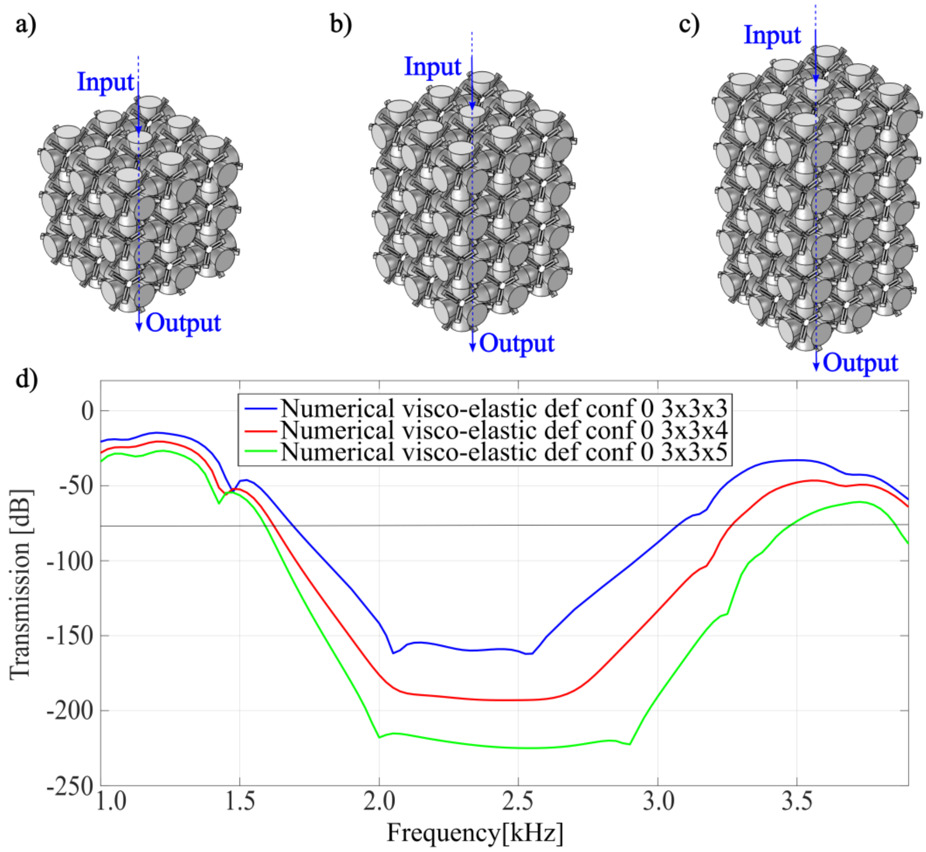

Figure s2: Transmission plot for periodic structures with an increasing number of layers in the propagation direction. a) 3x3x3, b) 3x3x4, c) 3x3x5 periodic structures and d) relative transmission plots. The thick black line represents the limit of the experimental setup.

3. Bandgap opening and closing frequencies

Bandgap regions can be customarily considered in periodic structures when attenuation is equal or lower than 40dB in the transmission spectrum. According to this hypothesis, the authors are able to determine the opening and closing frequencies of the bandgap for both undeformed and deformed configurations in a deterministic way (see crosses in Fig. s3). The values here extracted are reported in Tab. 2 of the paper.

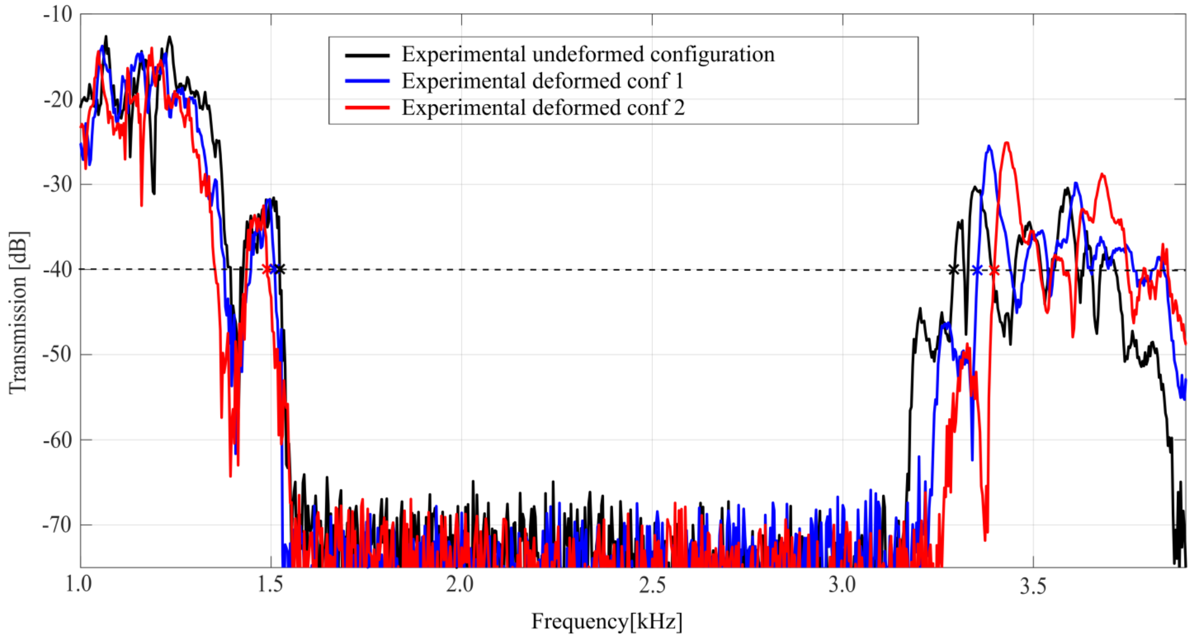

Figure s3: Experimental transmission spectra for both the undeformed and deformed configurations. The opening and closing frequencies of the first band gap are determined referring to the 40 dB level of attenuation.

4. Three-dimensional tuning of the bandgap of the proposed structure

Due to the good agreement between numerical and experimental results reported in Fig. 7 of the paper, the three-dimensional tuning of the first bandgap is shown through numerical simulations via COMSOL Multiphysics v5.3. Transmission spectra are shown in Fig. s4.

In Fig. s4 a)-b) the transmission spectra for both the undeformed and the deformed configurations along the two principal directions orthogonal to the tuning load direction are shown (associated to the Γ-Y path of the IBZ shown in Fig. 3 of the paper), Fig. s4 a) reports the same numerical curves as the Fig. 7 of the paper. In Fig. s4 c) the transmission spectra along the direction parallel to the tuning load direction (associate to the Γ-X path of the IBZ shown in Fig. 3 of the paper) are reported.

Finally, the transmission spectra for both the undeformed and deformed configuration computed by applying the input and measuring the output along two orthogonal directions are reported in Fig. s4 d) to show from a different perspective, and also less unit cells, the 3D tuning of the bandgap.

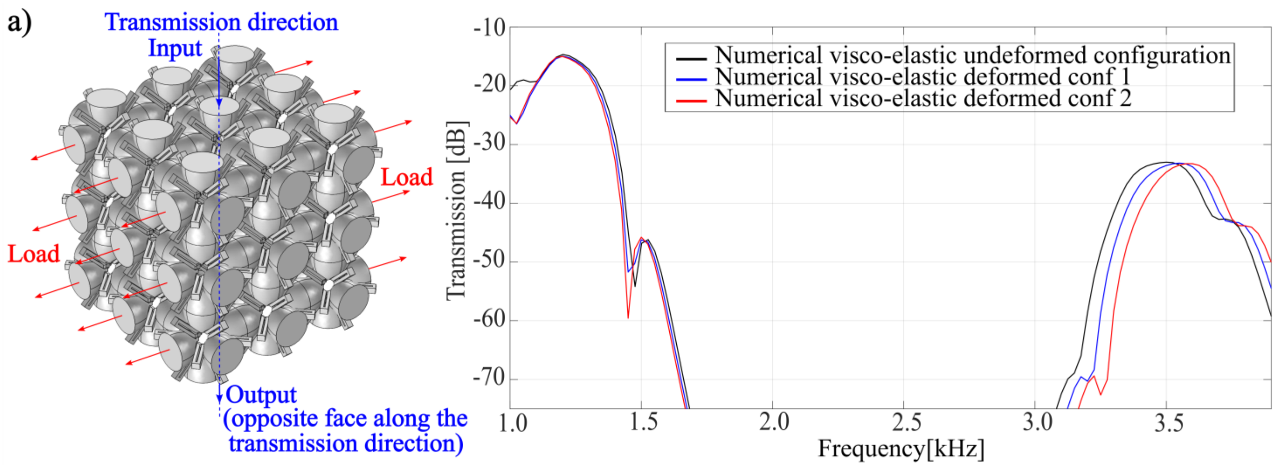

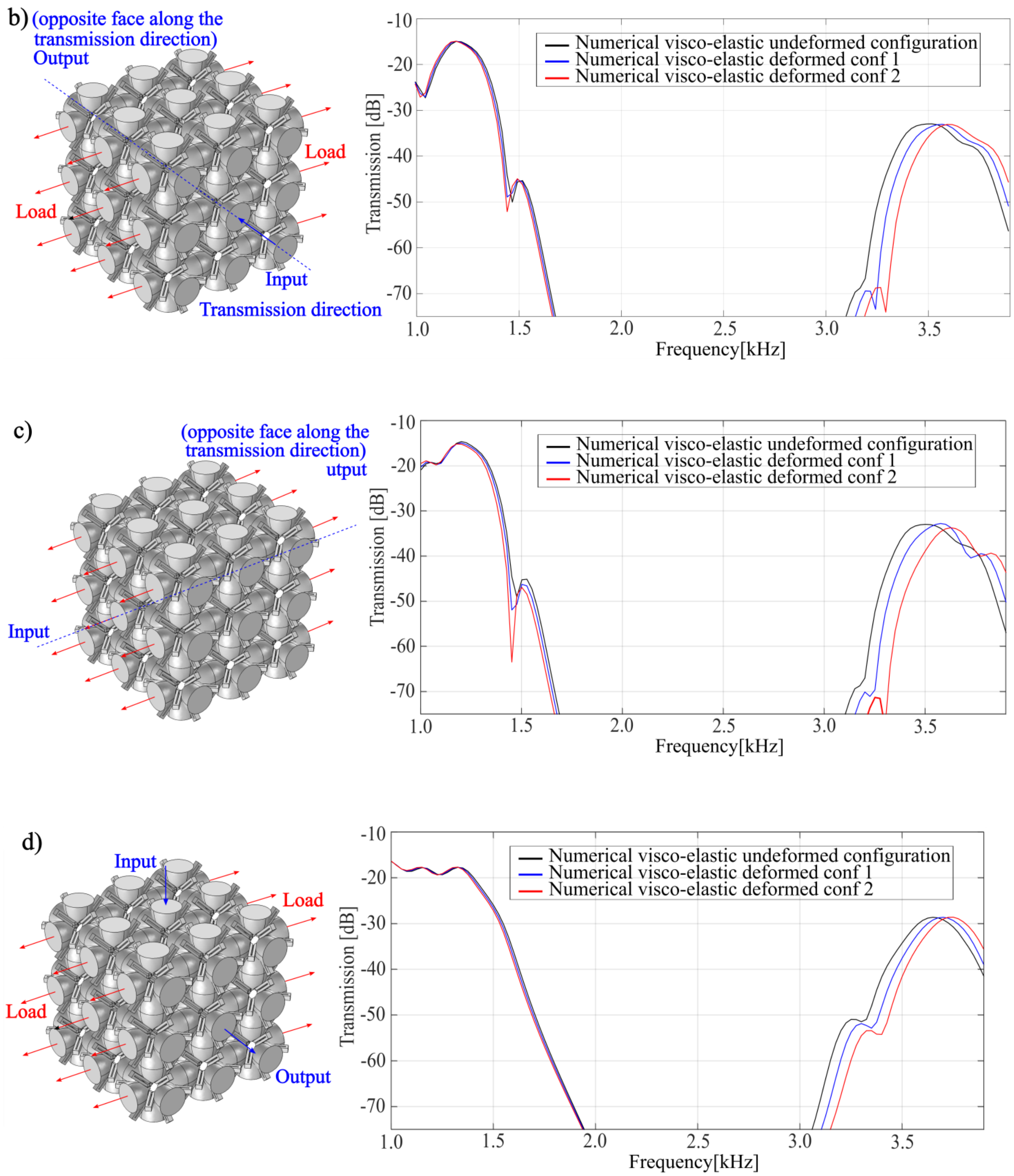

Figure s4: Three-dimensional tuning of the first bandgap. Transmission spectra simulated in COMSOL Multiphysics v5.3 along: a)-c) the three principal orthogonal directions and d) a different path in the IBZ.
